# Determinants of Poor Mental Health of Medical Students in Portugal—A Nationwide Study

**DOI:** 10.3390/healthcare11141991

**Published:** 2023-07-10

**Authors:** Vânia D’Alva-Teixeira, Maria Picó-Pérez, Pedro Morgado

**Affiliations:** 1 Life and Health Sciences Research Institute (ICVS), School of Medicine, University of Minho, Campus de Gualtar, 4710-057 Braga, Portugal; a90749@alunos.uminho.pt (V.D.-T.); mariapico231@gmail.com (M.P.-P.); 2 ICVS/3B’s, PT Government Associate Laboratory, 4710-057 Braga, Portugal; 3 Departamento de Psicología Básica, Clínica y Psicobiología, Universitat Jaume I, 12071 Castelló de la Plana, Spain; 4 2CA-Braga Cinical Academic Center, Hospital de Braga, 4710-243 Braga, Portugal

**Keywords:** distress, anxiety, depression, burnout, medical student, medical school

## Abstract

Medical students are a population that is vulnerable to the development of anxiety, depression, and burnout. This observational cross-sectional study sought to assess the levels of distress and identify precipitating factors in all students enrolled in a Portuguese medical school during the academic year of 2022/23. Students (n = 768) were surveyed via validated instruments to measure anxiety, depression, and burnout. Sociodemographic information was also collected through a questionnaire. The study indicated that almost half of this population had depressive symptoms. No differences were found in distress levels between medical schools, and when comparing curricular years, higher levels of distress were found in the pre-clinical years in comparison to the clinical ones. Burnout, being a woman, the existence of physical health problems, homo- and bisexual orientations, affective family problems, problems with relationships in the academic community, difficulties in academic performance, and daily organization were identified as predictors of distress. On the other hand, satisfaction with the social support received and with academic ratings were identified as protective factors. In conclusion, there is a high prevalence of distress in medical students, which is associated with personal, physical, social, economic, and academic factors. The identification of predictive factors of distress may allow for the early identification of vulnerable students and for intervention and prevention strategies to be defined.

## 1. Introduction

Medical students are a population at an increased risk of developing mental illness [1,2,3]. International studies estimate that one in three students suffers from depression, anxiety, or burnout, and this number can reach up to 50% [4,5,6,7,8]. In Portugal, the data are similar and equally worrying [9,10]. Despite the high levels of distress, the number of students who resort to seeking professional help is low. This leads to a clinical underdiagnosis with important consequences [11,12]; there is a well-established relationship between distress and decreased academic performance, substance abuse, and suicide [5,9,13,14,15]. Without treatment, symptoms can persist or worsen during clinical practice, leading to repercussions on the quality of healthcare provided: clinicians in distress show lower levels of empathy and professionalism [16,17,18], and there is also an increase in the number of adverse events, the mortality rate of patients, and the abandonment of one’s medical career [19,20,21]. Thus, the importance of early recognition of distress and its triggering factors in this population becomes clear [10].

Psychological distress, commonly just called distress, is a term that encompasses nonspecific symptoms of anxiety, depression, and burnout [22]. The use of this concept allows us to assess emotional distress in more depth through the characterization of symptoms that typically coexist with each other [23]. The high prevalence of distress has multifactorial causes, and the literature suggests that its associated risk factors are established even before medical education [24]. The path to medical training is guided by commitment, dedication, and, often, a sense of vocation that seems, in some cases, to translate into an increase in academic pressure exerted by the self and others during the process of obtaining one’s degree [24,25]. Course demands, workload, isolation, irregular sleep patterns, impostor syndrome, and maladaptive perfectionism are some of the intrinsic and extrinsic factors often found and synergistically associated in this population, enhancing the development of distress [26,27].

There are several studies, both national and international, that explore the prevalence of distress in the medical field; however, most of them focus only on one pathology and/or are carried out in small samples, making it difficult to assess the broader implications of this problem.

The present study addresses three dimensions of distress and explores potentially related factors in students who attended Portuguese medical education in the academic year of 2022/2023. With this comprehensive analysis of the mental health of medical students, we aim to expand the knowledge regarding the unique characteristics that make this sample vulnerable, and to provide crucial tools that will help medical schools to review intervention strategies and develop early detection plans.

## 2. Materials and Methods

### 2.1. Study Design

A cross-sectional analytical observational study was developed by researchers from the School of Medicine of the University of Minho in Portugal.

### 2.2. Study Participants

All students enrolled in the Integrated Master’s degree in Medicine (MIM) program in one of the nine Portuguese medical schools with a complete cycle of studies in the curricular year of 2022/2023 were invited to participate, with this being the only inclusion criterion. Students from the Faculty of Medicine of Catholica Medical School did not participate in the study due to refusal by the respective director.

### 2.3. Data Collection Process

Students were surveyed through an electronic version of a self-report questionnaire (through Google Forms). The questionnaire was shared via institutional email, social networks of the National Association of Medical Students (ANEM), and in the respective academic year groups. Data collection took place over three weeks (between 23 September 2022 and 13 October 2022).

Participants gave their informed consent before data collection; participation was free and they received no compensation for participating.

### 2.4. Ethical Considerations and Confidentiality

This study was approved by the Ethics Committee of the University of Minho (Braga, Portugal). Data confidentiality was guaranteed throughout the study.

### 2.5. Assessment Instruments

#### 2.5.1. Sociodemographic Questionnaire

To better characterize our sample, we included a sociodemographic questionnaire with 24 questions regarding social, academic, and financial factors, as well as perceived problems and distress-inducing events in the participant’s life (Supplementary Table S2).

#### 2.5.2. Beck Depression Inventory

The Beck Depression Inventory (BDI) is a scale composed of 21 questions in a self-report format created by Beck and adapted for the Portuguese population by Vaz Serra [28,29]. It encompasses the affective, cognitive, motivational, and physical components of depression and allows for the characterization of symptom intensity with a score from 0 to 63 points [30]. Its cut-off points are the following: no depressive symptoms (up to 12 points), mild depression (between 13 and 18 points), moderate depression (between 19 and 24 points), and severe depression (≥25 points).

#### 2.5.3. State-Trait Anxiety Inventory—Y Form

The State-Trait Anxiety Inventory—Y (STAI-Y) form was developed by Spielberg and translated for the Portuguese population by Danilo Silva [30]. It is composed of two self-assessment scales with 20 questions each. One is STAI-Y1 (or State anxiety), which allows for the identification of anxiety as a transitory state related to the feelings of the present moment, and the other is STAI-Y2 (or Trait anxiety), which characterizes anxiety as a stable state that is part of the individual’s personality traits and assesses commonly felt symptoms [31,32]. The answers range from “not at all” to “very much”, and the total score ranges from 20 to 80 points, with higher scores being associated with a state of anxiety or a more pronounced anxious trait. The 75th percentile was used as a cut-off (scores ≥ 52/54 points in STAI-Y1 and STAI-Y2, respectively) to identify cases with severe symptoms [10].

There are 10 inverted rating items in STAY-Y1 (corresponding to questions 1, 2, 5, 8, 10, 11, 15, 16, 19, and 20) and 9 in STAI-Y2 (questions 21, 23, 26, 27, 30, 33, 34, 36, and 39). The STAI-Y1 scale was applied first to prevent interference with the provided responses [32].

#### 2.5.4. Maslach Burnout Inventory Student Survey

The Maslach burnout inventory student survey (MBI-SS) is an inventory adapted for students by Shaufeli and for the Portuguese population by Maroco [32,33]. This scale consists of 15 questions and assesses 3 dimensions of burnout: emotional exhaustion (5 questions, corresponding to numbers 1, 4, 10, 13, and 7), disbelief (4 questions, corresponding to numbers 5, 2, 11, and 14), and academic effectiveness (6 questions, corresponding to numbers 15, 6, 8, 12, 9, and 3). In the present study, the term “academic ineffectiveness” was used through the inversion of the “academic effectiveness” subscale [33].

The MBI-SS does not allow for an overall burnout score. High scores for emotional exhaustion and disbelief and low scores for academic effectiveness have been found to be consistent with burnout. The 75th percentile was used as the cut-off point for each of the three dimensions [10].

### 2.6. Internal Consistency

All questionnaires showed satisfactory internal consistency (Cronbach’s alpha > 0.7) (Supplementary Table S1) [34].

### 2.7. Statistical Analysis

Statistical analysis was performed using the IBM^®^ SPSS^®^ Statistics software, version 28. A significance level of *p* < 0.05 with a confidence interval of 95% was established.

Considering the sample size (n > 30), and through observation of the histograms, an assumption of normality was made [35].

Differences in the curricular year and medical school among the BDI and STAY scores were compared by one-way analysis of variance (ANOVA), along with post hoc Tukey’s honestly significant difference test.

Linear regressions to identify predictors of depression and anxiety were performed. The different possible predictors of distress (or variables) were grouped into five blocks, (Supplementary Table S2) each corresponding to a different model. During the statistical analysis, variables with a binary response (yes/no) were summed and recoded to “no response checked/no problem” = 0, and “one or more response/problem” = 1. On the other hand, Likert variables were recoded to a scale of 0 to 100, then summed and recoded to “Not at all” = 0, “A little” = 1, “Average” = 2, and “A lot” = 3. To obtain the new variable “Burnout”, questions corresponding to each of the three scales were added and then recoded into a new one, where all values below the 75th percentile were defined as “No burnout” = 0 and all values equal or above as “Burnout” = 1.

## 3. Results

### 3.1. Personal, Sociodemographic, Economic, and Academic Factors

A total of 767 students participated in the study, and the general characterization of the sample is detailed in Supplementary Table S3. In general, the students’ level of satisfaction concerning their grades was lower than the level of satisfaction felt by their parents. Despite this, most participants were satisfied with the grades which they obtained (Supplementary Table S4).

The “Organization of academic work” issue appeared as the most frequent problem reported by students from all the included schools. “Physical health”, “Academic performance”, and “Money management” were other prevalent problems among the sample (Supplementary Table S5).

The existence of a “serious illness in someone close” in the last six months was the most often experienced negative event (Supplementary Table S6).

Regarding social and emotional support, most students had not resorted to psychological support since they started the course and were satisfied with the level of support they received from their social relationships (Supplementary Tables S7 and S8).

Finally, the socioeconomic data from the sample revealed that, in most cases, both parents of the students were in an active professional situation, and that there is no need to obtain financial aid (Supplementary Table S9).

### 3.2. Prevalence of Anxiety, Depression, and Burnout

The analysis of the total sample indicated that 46.9% of the students presented a BDI score compatible with the presence of depressive pathology (BDI > 12). Tiredness appeared as the most reported symptom, with 59.8% of students indicating that they felt “tired more easily than before”. Suicidal ideation was present in 12% of the surveyed students.

Regarding anxiety, 29.2 and 28.3% of the participants scored above the cut-off points for state and trait anxiety, respectively. Concerning burnout, the prevalence rates of emotional exhaustion, disbelief, and academic ineffectiveness were 27.4, 28.2, and 29.2% (Table 1 summarizes the prevalence of distress and Supplementary Table S10 the mean levels of distress).

The Pearson’s correlation test revealed that there is a strong association between state anxiety and trait anxiety (r > 0.50 in *p* < 0.001). For this reason, all subsequent analyses were focused on state-anxiety.

### 3.3. Distress per Curricular Year

The analysis of the entire sample showed that second-year students were the ones with higher levels of depression and emotional exhaustion (Table 2 and Supplementary Tables S11 and S12). On the other hand, sixth graders, followed by fifth graders, showed the lowest levels of depression.

### 3.4. Distress per Medical School

The one-way ANOVA comparing the means of depression, anxiety, and burnout for the different medical schools showed no significant differences between groups (Supplementary Table S13).

### 3.5. Predictive Factors of Depression

The analysis showed that all of the tested models were significantly correlated (*p*-value < 0.05) with the dependent variable (Supplementary Table S14). Burnout and social and financial factors made the largest contribution, with influences of 52.5% and 47.6% on depression.

The variables with the greatest influence on the prevalence of depression were, in descending order, burnout (β = 0.237; *p*-value < 0.001), the existence of physical health problems (β = 0.185; *p*-value < 0.001), sexual orientation (β = 0.133; *p*-value < 0.001), problems within the academic community (β = 0.121; *p*-value < 0.001), and family and affective problems (β = 0.102; *p*-value < 0.001) (Table 3). This study indicates that a non-heterosexual sexual orientation is associated with a higher level of depression.

Among the independent variables with an inverse relationship with depression, social support, followed by satisfaction with academic classification, had the greatest impact (β = −0.308; *p*-value < 0.001) and (β = −0.121; *p*-value < 0.001), showing that the greater the satisfaction with the support received and with academic classifications, the lower the prevalence of depression. The linear regressions results also showed being a woman to be associated with a higher prevalence of depression.

### 3.6. Predictive Factors of Anxiety

The analysis which we performed showed that all models, except the one that included academic factors, had a significant influence on the dependent variable (Supplementary Table S15).

The study shows that students experiencing burnout have a high risk of anxiety symptoms (β = 0.140; *p*-value < 0.001) (Table 4).

## 4. Discussion

This study demonstrated the existence of depressive symptoms in 46.9% of students. Although this figure is above the 20 to 30% which is frequently reported [4,5,10], it is in line with previous reports [2,36]. Suicidal ideation was reported in 12% of students, while studies focusing on this topic have shown a dispersion of values ranging from 6 to 50% [14,37,38].

Regarding anxiety, international studies point to average levels higher than those obtained in this study [6,39]. Anxiety symptoms are often associated with fatigue and academic overload, which is felt especially around evaluation periods [40]. The fact that the data were collected shortly after the beginning of the academic year may explain this result.

Regarding burnout, the small number of studies on the Portuguese population makes any comparisons impossible; even so, it was possible to infer that its existence is associated with a higher prevalence of depression and anxiety.

Distress values in the study population, although high and worrying, are not new and have been extensively described in the literature. The focus of this study was to identify and understand the factors behind them and to expand the existing literature. In this sense, the following dimensions were addressed and will be discussed here: personal, physical, social, economic, and academic.

In the personal dimension, this study showed a strong association between being a woman and the prevalence of depression, reinforcing what has previously been described [8,41]. The symptomatic expression of psychiatric pathology in men tends to be external, while in women, it is often internal [42,43]. This may be a cause of underdiagnosis, explaining the sex/gender disparity. It was not possible to establish relationships between distress and non-binary genders due to their reduced representation in the sample.

In our study, non-heterosexual students were also more depressed [44,45]. Minorities in general, and sexual minorities, have been described as a population which is more vulnerable to distress [44,45]. The challenges of establishing an identity with the pre-established “pattern” associated with the risks and consequences of segregation as a confounder may be one of the underlying factors.

Medical schools and society must seek to play an active role in the protection and, above all, the inclusion of minorities. This can be accomplished through education about unconscious bias and increased representation within the university’s workforce.

In the physical dimension, there are several factors to consider. The study showed that students with more physical health problems have more depressive symptoms. At the same time, a large percentage of students reported feeling more tired than usual. The association between distress and physical health problems has been described previously [40,46]. Here, there is the risk of establishing and perpetuating a cycle with distress symptoms created by physical symptoms and vice versa. Although these data were not included in this study, in the future, it may be important to explore the impact that regular physical exercise, sleep quality, and diet have on students’ mental health.

In the social dimension, it appears that a considerable percentage of students are not satisfied with their social activities or with the level of support received through their relationships. The demand experienced in medical school can mean a reduction in close ties and a feeling of isolation [46]. This is worrying, as the existence of a good social support network and satisfaction with social activities have been described as coping tools for academic challenges and aid in the prevention of distress [10,40,47].

An opportunity for intervention by medical schools arises here by encouraging participation in extracurricular activities and creating a medical curriculum that considers the level and duration of periods of greater academic load.

Regarding the financial dimension, most students reported not needing financial support, and our model did not show any significant association between this and the dimensions of distress under study. The route leading to medical school selects financially privileged students [48]. In addition, the financial burden required for higher education in Portugal is very different from that felt in other Western countries. Still, there is an established association between the existence of financial difficulties and higher levels of depression [49]. This should be anticipated by medical schools through structures to assist students in need.

Regarding the academic dimension, there was a discrepancy concerning which of the curricular years were associated with the highest levels of depression and anxiety. Some studies have indicated that distress increases in the first two years and decreases throughout the degree program [10,50]. Our work corroborates these results, showing that despite the high levels of anxiety experienced in the first year, the highest levels of distress are present in the second year, and the lowest in the sixth year. This may be due to the progressive development of a functional study method and academic load management tools. This would also explain why the pre-clinical years are characterized by higher levels of anxiety. This study, in agreement with others, demonstrates that difficulties associated with academic performance and daily organization have an impact on the prevalence of depression [45,51]. The early identification of students with difficulties in this field and the existence of programs that promote the sharing of advice among older students at different stages of training could have a positive influence on the students’ levels of distress in the first few years.

It is important to mention the association between satisfaction with ratings and depression, which was verified in this study, and the advantages and disadvantages associated with this relationship. If, on the one hand, it is positive to confirm that the feeling of reward for effort is a protective factor against distress, on the other hand, it is necessary to pay attention to the potentially dangerous association between academic validation and self-worth [52]. Our study demonstrates that the level of satisfaction that students feel for their academic performance is lower than their perception of the level of satisfaction felt by their parents. This may be due to the perfectionist and self-critical profile often associated with this sample [53]. This profile can impact students’ self-esteem, with consequences on levels of empathy and the clinical relationships established with patients [54]. It is important to provide students with an environment where there is room for error and where it is possible to explore other aspects of their personal and academic identity. This can be accomplished by diversifying the areas which are valued in the curriculum and adjusting the academic load.

Our study participants are in a highly demanding, but also honorable career, and we know that stress and perfectionism have been important factors in the pathways that brought them here. We focused on the negative elements of distress to alert authorities and encourage change because we believe that these often pass unnoticed. With that in mind, we look forward to exploring other perspectives on stress in future studies, and perhaps even how it can be balanced to maximize the positive elements.

Although each medical school has a particular and differentiated curriculum, no differences were found in their students’ distress levels. It is not clear why this occurs. It is possible that the similarities between schools are greater than their differences, or that an effective formula has not yet been developed to significantly reduce the levels of anxiety, depression, and burnout experienced by students. In addition, there is a stress factor common to all schools—the requirements of the path to accessing a specialty after graduation. There is work still to be done that extends beyond the training period, beyond the role of medical schools, and that concerns the prevailing culture that promotes the doctor to the role of “hero”, often describing him/her as “resilient” [55]. This idea may force future physicians into roles where there is little room to accommodate the physical, emotional, and psychological ups and downs that are part of the human experience [56].

### Study Limitations

The study was carried out with a convenience sample, so it is difficult to guarantee the true representation of Portuguese medical students. There may be an overestimation of the results because the greater number of responses may be coming from people to whom the topic being studied is more appealing (for example, students with symptoms of distress).

The study was conducted after a long period of vacation, shortly after the beginning of the academic year, which is another possible limitation.

The self-report format of the applied questionnaires is also a limitation of the study.

The lack of literature on distress in the Portuguese medical student population limits the number of comparisons that can be made, as well as the framing of the results.

## 5. Conclusions

The high prevalence of depression, anxiety, and burnout in medical students is a well-known problem in the medical community. The causes are multiple, and include factors of personal, physical, social, economic, and academic nature.

This study sought to understand the predictors associated with levels of distress and to identify some points of resolution in them that can be implemented by medical schools.

It is essential to identify and protect this vulnerable group of students and to rethink the characteristics of medical education and culture that enable the development of distress.

## Figures and Tables

**Table 1:** Prevalence of distress among Portuguese medical students in the academic year 2022/2023.

		Frequency, n (%) Total Sample	Frequency, n (%) Females	Frequency, n (%) Males
Depression	No depression (0 to 12)	407 (53.1)	338 (52.5)	65 (56.5)
	Mild (13 to 18)	146 (19.0)	121 (18.8)	23 (20.0)
	Moderate (19 to 23)	109 (14.2)	92 (14.2)	15 (13.9)
	Severe (≥24)	105 (13.7)	93 (14.4)	12 (10.4)
Anxiety (cut-off P75)	State anxiety	224 (29.2)	194 (30.12)	29 (25.2)
	Trait anxiety	217 (28.3)	191 (29.7)	24 (20.9)
Burnout	Emotional exhaustion	210 (27.4)	176 (27.3)	33 (28.7)
	Disbelief	216 (28.2)	176 (27.3)	37 (32.2)
	Academic ineffectiveness	224 (29.2)	179 (27.8)	44 (38.3)

**Table 2:** Average levels of depression, anxiety, and burnout.

	1st		2nd		3rd		4th		5th		6th	
	*M*	*SD*	*M*	*SD*	*M*	*SD*	*M*	*SD*	*M*	*SD*	*M*	*SD*
Depression	14.44	10.078	**16.92**	11.828	12.60	9.119	14.59	11.922	13.20	10.322	11.99	10.161
Anxiety	**49.41**	5.349	49.01	5.926	48.61	5.169	48.49	5.412	48.44	5.435	48.97	4.761
Emotional exhaustion	16.36	6.656	**18.32**	6.540	16.59	6.543	16.58	6.644	17.12	6.888	15.69	6.917
Disbelief	8.53	6.168	**9.77**	5.920	8.36	5.254	9.06	5.799	**9.77**	6.193	9.07	6.090
Academic ineffectiveness	19.62	5.653	19.41	5.732	**21.19**	6.169	20.44	6.172	01.20	5.636	21.10	5.815

The values in **bold** stand for statistically significant differences (*p* < 0.05).

**Table 3:** Linear regression coefficients for depression.

		B (Non-Standardized Coefficients)	β (Standardized Coefficients)	t	*p*-Value
1	Sex/gender	−1.801	−0.086	−3.149	**0.002**
	Age	−0.205	−0.046	−1.148	0.251
	Medical school	0.028	0.006	0.242	0.809
	Curricular year	0.099	0.016	0 400	0.689
	Sexual orientation	2.370	0.133	4.942	**<0.001**
2	Physical health	3.976	0.185	6.814	**<0.001**
	Substance abuse	−0.422	−0.009	−0.362	0.717
3	Satisfaction with academic rankings	−0.030	−0.121	−4.185	**<0.001**
	Difficulties associated with academic performance	1.524	0.071	2.378	**0.018**
	Daily organization	1.520	0.059	2.263	**0.024**
	Problems associated with relationships in the academic community	2.888	0.121	4.312	**<0.001**
4	Family and affective problems	2.199	0.102	3.925	**<0.001**
	Financial problems	0.616	0.028	1.089	0.276
	Social support	−0.048.	−0.308	−10.854	**<0.001**
5	Burnout	5.878	0.237	8.782	**<0.001**

The values in **bold** stand for statistically significant differences (*p* < 0.05).

**Table 4:** Linear regression coefficients for anxiety.

		B (Non-Standardized Coefficients)	β (Standardized Coefficients)	t	*p*-Value
1	Sex/gender	−0.288	−0.028	−0.716	0.050
	Age	0.188	0.085	1497	0.602
	Medical school	0.014	0.006	0.176	0.940
	Curricular year	−0.378	−0.123	−2161	0.321
	Sexual orientation	−0.925	−0.105	−2740	**0.035**
2	Physical health	0.627	0.059	1526	0.180
	Substance abuse	−0.704	−0.031	−0.860	0.546
3	Satisfaction with academic rankings	0.001	0.012	0.282	0.112
	Difficulties associated with academic performance	−0.009	−0.001	−0.020	0.894
	Daily organization	−0.388	−0.030	−0.820	0.452
	Problems associated with relationships in the academic community	−0.142	−0.012	−0.302	0.635
4	Family and affective problems	0.300	0.028	0.762	0.536
	Financial problems	0.355	0.033	0.893	0.742
	Social support	0.014	0.178	4422	0.102
5	Burnout	1.729	0.140	3671	**<0.001**

The values in **bold** stand for statistically significant differences (*p* < 0.05).

## Data Availability

Not applicable.

## Supplementary Materials

The following supporting information can be downloaded at: https://www.mdpi.com/article/10.3390/healthcare11141991/s1, Table S1: Internal consistency of the instruments used; Table S2: Independent variables grouped in models; Table S3: General characterization of the sample; Table S4: Descriptive analysis of the perception of academic performance; Table S5: Descriptive analysis of the perception of problems and difficulties; Table S6: Descriptive analysis of negative life events in the last six months; Table S7: Descriptive analysis of the use of psychological support resources; Table S8: Descriptive analysis of the perception of social support; Table S9: Descriptive analysis of the socioeconomic situation; Table S10: Mean distress scores; Table S11: Analysis of distress according to the curricular year (one-way ANOVA); Table S12.1: Comparison of anxiety level by curricular year (one-way ANOVA and post-hoc HSD Tukey); Table S12.2: Comparison of anxiety level by curricular year (one-way ANOVA and post-hoc HSD Tukey); Table S12.3: Comparison of anxiety level by curricular year (one-way ANOVA and post-hoc HSD Tukey); Table S13: Analysis of distress as a function of medical school (One-way ANOVA); Table S14: Values referring to linear regressions for the dependent variable of depression; Table S15: Values referring to linear regressions for the dependent variable of anxiety.
