# Supplementary material for: Determinants of Poor Mental Health of Medical Students in Portugal—A Nationwide Study
Source: Healthcare (Basel). 2023 Jul 10;11(14):1991. doi: 10.3390/healthcare11141991 (PMC10379586; doi:10.3390/healthcare11141991)
Supplement: Supplementary file 1 [file healthcare-11-01991-s001.zip › healthcare-2436193-supplementary.pdf]

Supplementary Table S1. Internal consistency of the instruments used

Instrument	Internal consistency Cronbach's alpha
BDI	0.919
STAI Y-1 (State Anxiety)	0.948
STAI Y-2 (Trait Anxiety)	0.952
MBI-SS (Emotional Exhaustion)	0.895
MBI-SS (Disbelief)	0.848
MBI-SS (Academic Ineffectiveness)	0,800

Supplementary Table S2. Independent variables grouped in models

Model	Independent variable created	Grouped independent variables
**1**	Sex/gender<br>Age<br>Medical school<br>Curricular year<br>Sexual orientation	
**2**	Physical health	Physical health problems<br>Serious illness or an accident in the last six months
	Substance abuse	Substance abuse: Alcohol<br>Substance Abuse: Tobacco
**3**	Satisfaction with academic rankings	Academic ratings in relation to effort expended<br>Level of satisfaction with academic ratings
	Difficulties associated with academic performance	
	Daily organization	Problems related to the organization of daily tasks<br>Problems related to the organization of academic work
	Problems associated with the relationship with elements of the academic community	Relationship with colleagues<br>Relationship with teachers
**4**	Family and affective problems	Relationship with family<br>Relationship with boyfriend<br>Death of a family member in the last six months<br>End of a stable love relationship in the last six months<br>Someone important has had a serious illness or accident in the last six months
	Financial problems	Difficulties associated with money management<br>Financial problems in the last six months
	Social support	Number of people nearby<br>Involvement and interest of close people<br>Level of satisfaction with social activities<br>Level of satisfaction with the support of the social relations network
**5**	Burnout	Emotional exhaustion<br>Disbelief<br>Academic ineffectiveness

Supplementary Table S3. General characterization of the sample

	Frequency (*n*)	Percentage (%)
**Age average**	21 years	
**Sex/Gender**		
Female	644	84.0
Male	115	15.0
Trans female	0	0
Trans male	3	0.4
Other	5	0.6
**Sexual orientation**		
Heterosexual	632	82.4
Bisexual	92	12.0
Homosexual	27	3.5
Other	14	1.8
**Curricular year**		
1st year	114	14.9
2nd year	158	20.6
3rd year	129	16.8
4th year	103	13.4
5th year	116	15.1
6th year	147	19.2

Supplementary Table S4. Descriptive analysis of the perception of academic performance

Variable	Frequency (*n*)	Percentage (%)
**Academic ratings in relation to effort spent**		
Lower to effort spent	311	40.5
In agreement with the effort spent	431	56.2
Superior to the effort expended	25	3.3
**Level of satisfaction with academic rankings**		
Dissatisfied	46	6.0
Not very satisfied	264	34.4
Satisfied	397	51.8
Very satisfied	60	7.8
**Parental satisfaction level regarding academic classifications**		
Dissatisfied	10	1.3
Not very satisfied	57	7.4
Satisfied	339	44.2
Very satisfied	361	47.1

Supplementary Table S5. Descriptive analysis of the perception of problems and difficulties

Variable	Frequency (*n*)	Percentage (%)
**Perception of problems or difficulties**		
Performance academic	342	44.6
Organization of daily tasks	287	37.4
Organization of academic work	559	72.9
Money management	274	35.7
Relationship with colleagues	197	25.7
Relationship with teachers	43	5.6
Relationship with family	171	22.3
Relationship with boyfriend	60	7.8
Physical health	374	48.8
Substance abuse: Alcohol	13	1.7
Substance Abuse: Tobacco	23	3.0
Substance abuse: Other drugs	6	0.8

Supplementary Table S6. Descriptive analysis of negative life events in the last six months

Variable	Frequency (*n*)	Percentage (%)
Death of a family member	123	16.0
End of a stable love relationship	97	12.6
Financial problems	153	19.9
Had a serious illness or an accident	37	4.8
Someone important in your life has suffered a serious illness or an accident	196	25.6

Supplementary Table S7. Descriptive analysis of the use of psychological support resources

Variable	Frequency (*n*)	Percentage (%)
Family doctor	78	10.2
Psychiatrist	134	17.5
Psychologist	284	37.0
I didn't use any of these resources	433	56.5

Supplementary Table S8. Descriptive analysis of the perception of social support

Variable	Frequency (*n*)	Percentage (%)
**Number of close people they trust**		
None	10	1.3
1 or 2	212	27.6
3 or 5	355	46.3
≥5	190	24.8
**Interest and involvement of close people**		
None	6	.8
Reduced	34	4.4
I'm not sure	40	5.2
Some	285	37.2
A lot	402	52.4
**Level of satisfaction with the support you get from your social network**		
Dissatisfied	33	4.3
Not very satisfied	223	29.1
Satisfied	385	50.2
Very satisfied	126	16.4
**Level of satisfaction with social activities**		
Dissatisfied	18	2.3
Not very satisfied	111	14.5
Satisfied	424	55.3
Very satisfied	214	27.9

Supplementary Table S9. Descriptive analysis of the socioeconomic situation

Variable	Frequency (*n*)	Percentage (*%*)
**Scholarship holder status**		
I need a scholarship, but I didn't get it	90	11.7
I have a scholarship	130	16.9
I am not a scholarship holder, nor do I need it	547	71.3
**Father's professional status**		
Active	670	87.4
Unemployed	16	2.1
Retired	63	8.2
Deceased	18	2.3
**Mother's professional status**		
Active	658	85.8
Unemployed	72	9.4
Retired	29	3.8
Deceased	8	1.0

Supplementary Table S10. Mean *distress scores*

	Average (*M*)	Standard Deviation (*SD*)	Min/Max scored	Scale Min/Max
Depression	14.00	10.73	0/62	0/63
State anxiety	48.84	5.35	29/68	20/80
Trait anxiety	50.39	5.75	20/70	20/80
Emotional exhaustion	16.82	6.73	0/30	0/30
Disbelief	9.12	5.92	0/24	0/24
Academic Ineffectiveness	20.29	5.88	4/35	0/36

Supplementary Table S11. Analysis of distress according to the curricular year (one-way ANOVA)

	F	*p* - value	*Effect size, η*
**Depression**	4.124	**0.001**	0.026
**Anxiety**	0.577	0.718	0.004
**Emotional exhaustion**	2.619	**0.023**	0.017
**Disbelief**	1.311	0.257	0.002
**Academic ineffectiveness**	2.252	**0.048**	0.015

The values in **bold** stand for statistically significant differences ($p < 0.05$).

Supplementary Table S12.1. Comparison of anxiety level by curricular year (one-way ANOVA and post-hoc HSD Tukey)

Dependent variable	Curricular year	Curricular year	Mean Difference	Standard Error	*Sig.*	Confidence Interval 95%	
						Inferior limit	Upper limit
**Depression**	**1st**	**2nd**	-2.479	1.306	0.404	-6.21	1.25
		**3rd**	1.842	1.366	0.758	-2.06	5.74
		**4th**	-0.0154	1.444	1,000	-4.28	3.97
		**5th**	1.240	1.401	0.950	-2.76	5.24
		**6th**	2.452	1.326	0.435	-1.34	6.24
	**2nd**	**2nd**	2.479	1.306	0.404	-1.25	6.21
		**3rd**	4.321	1.261	**0.008***	0.72	7.92
		**4th**	2.325	1.346	0.513	-1.52	6.17
		**5th**	3.719	1.299	**0.049**	0.01	7.43
		**6th**	4.931	1.218	**<0.001**	1.45	8.41
	**3rd**	**2nd**	-1.842	1.366	0.758	-5.74	2.06
		**3rd**	-4.321	1.261	**0.008**	-7.92	-.072
		**4th**	-1.995	1.404	0.714	-6.01	2.02
		**5th**	-0.0601	1.360	0.998	-4.49	3.28
		**6th**	0.611	1.282	0.997	-3.05	4.27
	**4th**	**2nd**	0.154	1.444	1.000	-3.97	4.28
		**3rd**	-2.325	1.346	0.513	-6.17	1.52
		**4th**	1.995	1.404	0.714	-2.02	6.01
		**5th**	1.394	1.438	0.928	-2.72	5.50
		**6th**	2.606	1.365	0.398	-1.29	6.51
	**5th**	**2nd**	-1.240	1.401	0.950	-5.24	2.76
		**3rd**	-3.719	1.299	**0.049**	-7.43	-0.01
		**4th**	0.601	1.360	0.998	-3.28	4.49
		**5th**	-1.394	1.438	0.928	-5.50	2.72
		**6th**	1.212	1.320	0.942	-2.56	4.98
	**6th**	**2nd**	-2.452	1.326	0.435	-6.24	1.34
		**3rd**	-4.931	1.218	**<0.001**	-8.41	-1.45
		**4th**	-0.611	1.282	0.997	-4.27	3.05
		**5th**	-2.606	1.365	0.398	-6.51	1.29
		**6th**	-1.212	1.320	0.942	-4.98	2.56

The values in **bold** stand for statistically significant differences ($p < 0.05$); $*p<0.01$

Supplementary Table S12.2. Comparison of anxiety level by curricular year (one-way ANOVA and post-hoc HSD Tukey) (continuation)

Dependent variable	Curricular year	Curricular year	Mean Difference	Standard Error	*Sig.*	Confidence Interval 95%	
						Inferior limit	Upper limit
**Anxiety**	**1st**	**2nd**	0.400	0.659	0.991	-1.48	2.28
		**3rd**	0.800	0.689	0.855	-1.17	2.77
		**4th**	0.927	0.729	0.800	-1.15	3.01
		**5th**	0.973	0.707	0.742	-1.05	2.99
		**6th**	0.439	0.669	0.986	-1.47	2.35
	**2nd**	**2nd**	-0.400	0.659	0.991	-2.28	1.48
		**3rd**	0.400	0.636	0.989	-1.42	2.22
		**4th**	0.527	0.679	0.971	-1.41	2.47
		**5th**	0.573	0.655	0.953	-1.30	2.45
		**6th**	0.040	0.614	1,000	-1.71	1.79
	**3rd**	**2nd**	-0.800	0.689	0.855	-2.77	1.17
		**3rd**	-0.400	0.636	0.989	-2.22	1.42
		**4th**	0.127	0.708	1,000	-1.90	2.15
		**5th**	0.173	0.686	1,000	-1.79	2.13
		**6th**	-0.360	0.647	0.994	-2.21	1.49
	**4th**	**2nd**	-0.927	0.729	0.800	-3.01	1.15
		**3rd**	-0.527	0.679	0.971	-2.47	1.41
		**4th**	-0.127	0.708	1,000	-2.15	1.90
		**5th**	0.046	0.726	1,000	-2.03	2.12
		**6th**	-0.0487	0.689	0.981	-2.46	1.48
	**5th**	**2nd**	-0.973	0.707	0.742	-2.99	1.05
		**3rd**	-0.573	0.655	0.953	-2.45	1.30
		**4th**	-0.173	0.686	1,000	-2.13	1.79
		**5th**	-0.046	0.726	1,000	-2.12	2.03
		**6th**	-0.533	0.666	0.967	-2.43	1.37
	**6th**	**2nd**	-0.439	0.669	0.986	-2.35	1.47
		**3rd**	-0.040	0.614	1,000	-1.79	1.71
		**4th**	0.360	0.647	0.994	-1.49	2.21
		**5th**	0.487	0.689	0.981	-1.48	2.46
		**6th**	0.533	0.666	0.967	-1.37	2.43

Supplementary Table S12.3. Comparison of anxiety level by curricular year (one-way ANOVA and post-hoc HSD Tukey) (continuation)

Dependent variable	Curricular year	Curricular year	Mean Difference	Standard Error	*Sig.*	Confidence Interval 95%	
						Inferior limit	Upper limit
**Burnout - EE**	**1st**	**2nd**	-1.957	0.823	0.166	-4.31	0.39
		**3rd**	-0.229	0.861	1.000	-2.69	2.23
		**4th**	-0.223	00,911	1,000	-2.82	2.38
		**5th**	-0.761	0.883	0.955	-3.28	1.76
		**6th**	0.666	0.836	0.968	-1.72	3.05
	**2nd**	**2nd**	1.957	0.823	0.166	-0.39	4.31
		**3rd**	1.727	0.795	0.252	-0.54	4.00
		**4th**	1.734	0.848	0.318	-0.69	4.16
		**5th**	1,196	0.819	0.690	-1.14	3.54
		**6th**	2.623	0.768	**0.009***	0.43	4.82
	**3rd**	**2nd**	0.229	0.861	1.000	-2.23	2.69
		**3rd**	-1.727	0.795	0.252	-4.00	0.54
		**4th**	0.007	0.885	1.000	-2.52	2.54
		**5th**	-0.532	0.857	0.990	-2.98	1.92
		**6th**	0.895	0.808	0.878	-1.41	3.20
	**4th**	**2nd**	0.223	0.911	1.000	-2.38	2.82
		**3rd**	-1.734	0.848	0.318	-4.16	0.69
		**4th**	-0.007	0.885	1.000	-2.54	2.52
		**5th**	-0.538	0.907	0.991	-3.13	2.05
		**6th**	0.889	0.861	0.907	-1.57	3.35
	**5th**	**2nd**	0.761	0.883	0.955	-1.76	3.28
		**3rd**	-1.196	0.819	0.690	-3.54	1.14
		**4th**	0.532	0.857	0.990	-1.92	2.98
		**5th**	0.538	0.907	0.991	-2.05	3.13
		**6th**	1.427	0.832	0.522	-0.95	3.80
	**6th**	**2nd**	-0.666	0.836	0.968	-3.05	1.72
		**3rd**	-2.623	0.768	**0.009***	-4.82	-.43
		**4th**	-0.895	0.808	0.878	-3.20	1.41
		**5th**	-0.889	0.861	0.907	-3.35	1.57
		**6th**	-1.427	0.832	0.522	-3.80	0.95

The values in **bold** stand for statistically significant differences ($p < 0.05$); *$p<0.01$

Supplementary Table S13. Analysis of distress as a function of medical school (One-way ANOVA)

	F	***p*** **- value**	***Effect size , η***
**Depression**	1.461	0.178	0.013
**Anxiety**	1.013	0.421	0.009
**emotional exhaustion**	1.215	0.292	0.011
**Disbelief**	0.969	0.453	0.009
**Academic ineffectiveness**	0.731	0.645	0.007

Supplementary Table S14. Values referring to linear regressions for the dependent variable depression

Model	Adjusted R square	*R*	ANOVA (*F*)	ANOVA (*p*- value)
**1**	0.057	0.252	10,316	**<.001**
**2**	0.209	0.465	27,199	**<.001**
**3**	0.370	0.616	35,385	**<.001**
**4**	0.476	0.697	31.900	**<.001**
**5**	0.524	0.731	43.492	**<.001**

The values in **bold** stand for statistically significant differences ($p < 0.05$); Model 1: Age, sex/gender, curricular year, medical school, sexual orientation; Model 2: Physical health; Model 3: Academic factors; Model 4: Social and financial factors; Model 5: Burnout

Supplementary Table S15. Values referring to linear regressions for the dependent variable anxiety

Model	Adjusted R square	*R*	ANOVA (*F*)	ANOVA (*p*- value)
**1**	0.012	0.134	2.792	**0.017**
**2**	0.012	0.146	2.360	**0.022**
**3**	0.011	0.160	1.797	0.051
**4**	0.030	0.219	2.702	**<0.001**
**5**	0.046	0.255	3.462	**<0.001**

The values in **bold** stand for statistically significant differences ($p < 0.05$); Model 1: Age, sex/gender, curricular year, medical school, sexual orientation; Model 2: Physical health; Model 3: Academic factors; Model 4: Social and financial factors; Model 5: Burnout
